# “I’m Not Sure We Had A Choice”: Decision Quality and The Use of Cardiac Implantable Electronic Devices In Older Adults With Cognitive Impairment

**DOI:** 10.26502/fccm.92920032

**Published:** 2018-02-12

**Authors:** Nicole R. Fowler, C. Elizabeth Shaaban, Alexia M. Torke, Kathleen A. Lane, Samir Saba, Amber E. Barnato

**Affiliations:** 1 Indiana University Center for Aging Research, Indianapolis, IN, USA; 2 Regenstrief Institute, Inc., Indianapolis, IN, USA; 3 Department of Medicine, Indiana University School of Medicine, Indianapolis, IN, USA; 4 Department of Epidemiology, Graduate School of Public Health, University of Pittsburgh, Pittsburgh, PA, USA; 5 Department of Biostatistics, Indiana University School of Medicine, Indianapolis, IN, USA; 6 Division of Cardiology, Department of Medicine, School of Medicine, University of Pittsburgh, Pittsburgh, PA, USA; 7 The Dartmouth Institute of Health Policy and Clinical Practice, Geisel School of Medicine at Dartmouth, Lebanon, NH, USA

**Keywords:** Alzheimer’s Disease, Cardiac Implantable Electronic Device, Decision Making, Dementia

## Abstract

**Background:**

The decision to implant a cardiac device in a person with Alzheimer’s disease or related dementia requires considering the possible trade-offs of quality of life (QOL) and quantity of life. This study measured the decision-making experience of patients with and without cognitive impairment (CI) who received a cardiac device and their family members who were involved in the decision.

**Methods and Results:**

Semi-structured interviews and questionnaires were administered with 15 patient-family member dyads. Interviews revealed few conversations between physicians, patients and family members about the patient’s cognitive status or about the benefits, risks, and long-term implications of the device for someone with CI. Participants largely stated that the decision to get the device was based on the patient’s functional status at the time of the implant, and not on expectations about future functioning. Patients with CI had more regret, measured with the Decision Regret Scale (DRS), (p=0.037) and family members of patients without CI reported more decisional conflict, measured with the Decisional Conflict Scale (p=0.057).

**Conclusions:**

Although CI impacts life expectancy and QOL, cognitive status was largely not discussed prior to device implant. Few differences were found between the experiences of dyads that included patients with or without CI.

## 1. Introduction

In the U.S., there are 5.5 million people with Alzheimer’s disease or a related dementia (AD) [1] and 6 million people with mild cognitive impairment (MCI) [2, 3]. The presence of AD and MCI are independent predictors of a shortened life expectancy [4–6]. Recent meta-analyses and large cohort studies indicate that people with AD have an average life expectancy of 4.5 years from diagnosis [4–8]. That is impacted by dementia subtype, patient age, and severity of impairment at diagnosis [4–8]. Family caregivers are frequently involved in medial decision-making for patients with AD regarding their medical care and treatment [9–12].

Most adults 65 and over have multimorbidity—the presence of two or more medical conditions, and this prevalence increases with age. Cardiac co-morbidities, such as coronary heart disease are common in older adults, including those with AD [13–15]. Moreover, some studies have found an association between atrial fibrillation and incident dementia, beyond its effect on stroke [16]. Among Medicare beneficiaries with AD, 29.7% have coronary heart disease, 27.8% have congestive heart failure (CHF), and 24.7% have cardiac dysrhythmias [17]. A potential treatment option for patients with these cardiac co-morbidities is the implantation of a cardiac implantable electronic device, such as a pacemaker or implantable cardioverter-defibrillator (ICD).

Age and functional status at the time of device implant impact patient’s mortality post-device [18–21]. Despite having shorter life expectancies and other co-morbidities, many older adults with cognitive impairment (CI) receive cardiac devices, some beyond the time that it is clinically beneficial [12]. This raises questions about the risks of device placement in patients with CI as they are twice as likely to be admitted to a nursing home immediately and require long-term use of nursing homes after receiving a cardiac device [22]. The decision to implant a cardiac device in a person with CI involves unique considerations about the possible trade-offs of quality of life (QOL) and quantity of life [23]. Following device implantation, death from AD, which has been characterized by some as a state worse than death, may be prolonged [24–26]. We are unaware of any prior studies addressing decision-making by patients with CI, or their family members, regarding cardiac devices. The goals of this study are to compare the decision-making experience of a cohort of patients with and without CI who received a cardiac device as well as a family member who was involved in the decision to get a cardiac device.

## 2. Methods

### 2. 1 Participants, setting, and procedures

We recruited participants from the University of Pittsburgh Alzheimer Disease Research Center (ADRC). All study procedures were approved by the Institutional Review Board (IRB) at the University of Pittsburgh.

Patients in this study were active ADRC participants with or without CI, ≥50 years of age, able to read and speak English, and had received a single or dual chamber pacemaker and/or ICD after their baseline ADRC visit between 2002 and 2013. Inclusion criteria for family members included those who were named as the primary caregiver for an ADRC patient with MCI or AD or named by the non-cognitively impaired patient as the person who helped them make a decision about the device implantation. Patients were excluded from the study if they were too cognitively impaired to participate in an interview. Family members were excluded if they reported not being involved in the decision about the device implantation.

We used a maximum variation sampling strategy for ADRC participants without CI and with varying levels of severity of cognitive impairment at the time of device implant [27]. Sampling for family members included someone for each living patient participant and for every deceased patient who met study criteria.

Interviews were conducted with all family members and the subset of patients who were still alive and cognitively able to participate to obtain socio-demographic information, questionnaire, and semi-structured interview data. Additional research and clinical data were collected from the ADRC research database and from the patients’ medical records.

### 2.2 Measures

Socio-demographic data collected from patient participants included age, sex, race, and ethnicity. For patients who had died, date of death was obtained from the ADRC research database and confirmed with the family member. For family members, we collected sex, ethnicity, race, and their relationship to the patient.

Cognitive, functional, and clinical data collected included the Mini-Mental State Examination (MMSE) score [28] and the Functional Activities Questionnaire (FAQ), [29] a measure of activities of daily living closest to the date of device implant. Additionally, we reviewed all of the patient’s medical records at any point during the encounter for when the device was implanted to verify the date of device implant, type of device, device vendor, indication for the device, ACC/AHA Classification noted by the implanting physician, and to determine if any diagnosis of CI (AD, MCI or other) was noted.

Questionnaire data focused on measuring known constructs of medical decision-making quality. To measure role in decision making, we used the Control Preferences Scale (CPS), which consists of one item with a five-point scale.[30] Patient and family participants were categorized as having an active, shared, or passive role in the decision regarding receipt of the cardiac device.

*We used the* Decisional Conflict Scale (DCS) [31] to measure patients’ and family members’ level of uncertainty about getting a cardiac device. The DCS contains three sub-scales: (1) uncertainty, (2) factors contributing to the uncertainty, and (3) perceptions of effective decision-making. The DCS includes 16 questions regarding a medical decision the participant has made or is about to make using a five-point Likert scale [31]. The range of scores for the DCS is 0 to 100, where higher scores indicate more conflict.

To measure regret regarding the decision to get a cardiac device, we used the Decision Regret Scale (DRS), [32] which measures “distress or remorse after a health care decision”.

The range of scores for the DRS is 0 to 100, where higher scores indicate more regret (and low satisfaction with the decision) [32–34]. Scores >25 are considered to reflect significant ambivalence about a decision and scores between 10–25 are defined as mild ambivalence [33].

Semi-structured interview data was collected from all enrolled family members and the seven patients who were still alive and cognitively able to participate, including five patients without CI and two patients with CI. Interviews were conducted in-person and over the phone and separately from the other dyad member. Interview questions were developed by the research team and piloted with the first two interviews. Two study team members (NRF and CES) conducted all in-depth interviews with questions that were broad and open-ended. We invited participants to relate their personal narratives regarding their experiences with decision-making about cardiac device implantation. The audio recordings were examined and transcribed, and no systematic differences were discerned between interviewers. Participants responded in an equally open and forthcoming way to both interviewers. To maximize the trustworthiness of our data collection, we continued recruiting participants until no new major themes emerged [35, 36].

### 2.3 Analysis

We conducted qualitative analyses of the interview data guided by the methods of grounded theory [36]. The steps included open coding, assigning topical codes to the text of the interviews to form a codebook, organizing themes, comparing the content of each new interview to the existing codebook categories and modifying the codebook accordingly and developing categories and defining the relationships among them and their possible implications [37].

To ensure reliability between the coders in identifying and assigning codes, each coder reviewed a random 10% sample of the transcripts coded by the other research team member. The crosschecking coder could either endorse or contest the existing coding. Following a consensus meeting of the coders, the contested codes were deleted, replaced, modified, or added to the codebook. Statistical analyses for the questionnaire data included descriptive analysis of the population, including means, standard deviations for continuous data and counts and percentages for categorical data. T-tests were used to compare the DRS and DCS scores of both patients and family members by the type of device and patient cognitive status at the time of implant.

## 3. Results

### 3.1 Characteristics of Participants

Twenty-five patients received a pacemaker or ICD during the observation period; 10 patients or family members declined to participate. Of the 15 patient-family member dyads who participated, all family members participated in an interview and completed questionnaires. Seven patients from the dyads were alive and cognitively able to participate in an interview and completed questionnaires. More patients with CI at the time of device implant were deceased or too impaired to participate at study enrollment than patients who were not cognitively impaired.

The mean age (SD) of the patients at the time of device implant was 80.9 (SD 5.1). Sixty percent were male, and 100% were white. At the time of device implant, eight patients had a diagnosis of AD, two had MCI, and five had no CI.

Nine patients received a pacemaker and six received an ICD. Twelve patients received a device for an AHA/ACA Class I indication defined as evidence and/or general agreement that the device would be beneficial, useful, and effective for the patient [12, 38, 39]. Six had sinus node dysfunction, four received a device for the primary prevention of sudden cardiac death (SCD), three for atrioventricular block, and two for secondary prevention of SCD.

Family members who participated in this study were mostly female (80%); eight were the spouse of the patient (53%), and seven (47%) were an adult child. Table 1 shows the descriptive data of the patient-family dyads.

### 3.2 Qualitative Findings

As shown in Table 2, three main themes emerged from the interviews: (1) the limited influence of the patient’s cognitive status in medical decision-making about cardiac co-morbidities and cardiac devices, (2) circumstances around the decision to get the device, and (3) discussions about the risks, benefits, and long-term implications of getting a cardiac device. Representative quotations for each of these themes from the patients and caregivers are shown in Table 2.

### 3.3 CI was Not a Factor in Decision Making

Few patients with CI or their family members reported having a discussion about the patient’s cognitive status with the physician. In all cases but one, it was unclear from the interviews and medical record reviews if the physician was even aware of the patient’s MCI or AD or if they believed that it was an important factor to be discussed. Only one out of ten patients with CI had a diagnosis or notation in the medical record. Because the patients and family members largely did not bring up the patient’s CI with the physician, there was no discussion about the impact of the device on prolonging life, improving QOL, or risks post-implantation in the setting of also having MCI or AD. Even in the one case where it was made explicit to the medical team about the patient’s AD, it was not discussed in the context of the cardiac device (Quote 1).

None of the patients with cognitive impairment or the family members of patients with cognitive impairment, reported discussing their cognitive status with the physician implanting the device or its role in the decision to get a device. As previously mentioned, the medical records largely lacked information on the patients’ cognitive status; it was noted in only one case. Although family members acknowledged the patient’s MCI or AD during the interviews, opinions were mixed as to whether the CI should influence the decision-making process for getting a cardiac device. Most had a difficult time articulating the impact of AD on life expectancy and its role in medical decision-making about cardiac co-morbidities, and many were unaware of the trajectory and prognosis of AD. Patients and family members largely stated that the decision to get the device was based on the patient’s functional status at the time of the implant; however, a majority of them could imagine a time when the patient’s CI would be too severe to get a device and acknowledged that they would focus on QOL only (Quote 2).

Regarding the future health status and the need for re-implantation or a new battery or device, all seven patients interviewed stated that they would make the same decision. However, half of them stated that they might reconsider based on their functional status at the time (Quote 3).

### 3.4 Circumstances around the decision to get the device

#### 3.4.1 Urgent Need

Both patients and family members perceived the decision to get the device as urgent. All patients received a device during an inpatient stay; no devices were placed electively, although many had a history of abnormal heart rhythm or cardiac disease (Quote 4).

#### 3.4.2 The Physician’s Recommendation

Many family members and patients believed that if the physician says the patient needs it, they must need it and there are no other options. Family members reported that the physician strongly advised that the patient get the device (Quote 5).

Regarding the role of the family member in decision-making, five stated that they went along with what the physician recommended without discussion, and ten reported that they were involved in the decision-making (Quote 6).

#### 3.4.3 Decision Regret

When asked about whether the decision to get a cardiac device was a “good” or “bad” decision, or if they had regret, some patients reflected that it was not a “good decision” (Quote 7). Some family members of patients with CI also reported reflecting on whether getting the device was a “good decision” (Quote 8).

#### 3.4.4 Not a Decision

An overwhelming majority of patients and family members did not acknowledge that getting the device was a decision and were strongly influenced by the presentation of information by the physician, especially regarding how getting it would impact symptom relief (Quote 9). In some cases, patients and family members reported that the physician described the implant of a cardiac device as “necessary” and would help the patient “feel better”. In other cases, patients and family members did not perceive getting the cardiac device as a choice, rather a decision that was made by their doctor and simply presented to them (Quote 10).

#### 3.4.5 QOL

Discussions with the patient’s physician tended to focus on the “necessity” of the device rather than the benefits and risks. Topics focused more on the benefits of getting the device, including enhanced QOL and symptom relief. Some family members thought or were told that that receipt of a device might have a positive impact on the patients’ cognitive function. Family members largely stated that the biggest factor was to improve the QOL for the patient (Quote 11).

#### 3.4.6 Risks, benefits and long-term implications of getting a cardiac device

Few family members and patients reported having a conversation about the benefits, risks, and long-term implications of getting the device. Many reported that they were unsure what types of questions to ask because they did not have any experience with cardiac devices (Quote 12).

Many family members reported wishing they would have discussed more about the risks and alternatives to getting a cardiac device (Quote 13). Family members also expressed interest in learning more information regarding the benefits of the device and if it would increase the patient’s life expectancy and what specific symptoms should improve after the device is implanted.

#### 3.4.7 Decision Quality Measures

Most participants described having an active role in the decision and levels of decisional regret and decisional conflict were low to moderate. While statistical tests should be interpreted cautiously in this small sample, there were some statistically significant differences between patients and families by patient cognitive status. Overall, patients had an average DRS score of 16.4 (SD 24.4, range 0–65) while the average DRS score for family members was 14.9 (SD 19.1, range 0–50). The average DRS score for patients with CI was 45 (SD 28.3), and for patients without CI, the average DRS score was 5 (SD 11.2) (p=0.03). There was no difference in DRS scores for patients who received a pacemaker vs. an ICD (p=0.12) or their family members (p=0.28). Patients had an average DCS score of 38.5 (SD 36.1, range, 3.1–100) while family members reported slightly lower DCS, with a mean DCS score of 38.2 (SD 24.9, range 1.6–100). Family members of patients without CI reported higher levels of decisional conflict at the time of the decision to implant a device compared to family members of patients with CI (DCS scores 57.8 vs. 30.3, *p*=0.06) although differences did not meet statistical significance in this small sample.

## 4. Conclusion

The present study was undertaken to describe and compare the decision-making experience of patients with and without CI and their family members regarding cardiac devices. While a variety of circumstances precipitated the decision to implant a cardiac device, most felt that there was no decision to make and were greatly influenced by their physician’s recommendations. All patients in this study met the indications for the device; yet, few of the indications were described as clinically urgent in the medical record, despite what some dyads reported.

The state of uncertainty about future events related to a decision is a key element of all decision-making, and decision makers often experience regret when decision outcomes, particularly poor outcomes, are compared with alternative outcomes had another option been chosen [38]. Even the most informed patients may have regret and psychological consequences if the outcomes of their treatment are not as expected or if treatment-related side-effects compromise their QOL [40]. However, this belief has not been assessed in adults who receive a cardiac device while already having CI or among their family members. Cognitive function and progression of AD affect prognosis and QOL of the patient and, therefore, should be incorporated into medical decision making. Circumstances in which CI co-occurs with other medical conditions will be increasingly common in our age of multimorbidity. The results of the present study suggest that CI is rarely incorporated despite the fact that functional status of the patient at the time of implant was a strong influence on decision-making. Indeed, most participants exhibited decision myopia [22] and had a difficult time articulating the relationship between having MCI or AD and its role in medical decision making about cardiac co-morbidities. Thus, further studies are required to analyze particular decision needs among patients with CI and their family caregivers.

Additionally, patients with no CI were more likely to report that they made the decision to get the device either alone or with their doctor. In many cases, the patient’s description of the process was congruent with that of the family member. Family members of patients with CI at the time of device implant were more likely to report their role in the decision as either active or shared, both with and without the patient’s involvement. Many patients and family members described the physician’s role as active. Although patients and family members did not overwhelmingly report feelings of regret about getting a device in the questionnaires, expressions from the sample were more conflicted in the interviews. Some patients and family members had regret or questioned whether the decision was “the right one.” However, few patients endorsed a sense of regret, which may be related to the fact that few patients and family members reported any adverse events as a result of getting the device, which has been correlated with regret [41, 42]. If faced with the decision to replace the battery, most patients and family members felt they would make the same decision again; however, many said that they might reconsider based on their functional status at that time.

Regarding medical decision-making, there are two main sources of conflict. The first is the inherent difficulty that arises from making decisions that have both positive and negative effects. The second source is from aspects of the decision that are modifiable (e.g., lack of knowledge about options and outcomes, unrealistic expectations, unclear values, unclear perceptions of those presenting information, social pressures, lack of support or mismatch between the preferred role in decision-making and the actual role, lack of skills and self-confidence to make the decision, and lack of other resources) [38]. In the present study, patients and family members reported limited discussion regarding their decision to get a cardiac device. There was little evidence that the physicians who were implanting the cardiac devices had knowledge of or engaged in discussions about the patient’s cognitive status; an indication of dementia was identified in only one medical record. This is consistent with previous research showing providers’ lack of awareness of patient’s cognitive status and a reluctance to have discussions about both the risks and benefits of device implantation and deactivation [41, 42]. Many noted that their physicians concentrated on the “necessity” of the device, and few family members felt that there was a real choice, deferring to the physician’s recommendations. This is consistent with previous studies about other cardiac devices, including left ventricular assist devices (LVADs) [43]. Some family members thought or were told that that receipt of a device might have an impact on the patients’ cognitive function; however, evidence is mixed to support this claim [45, 46]. The family members’ lack of informed thinking about the implications of the device on longer-term quantity and QOL in their loved one with CI, as well as a lack of competing death risks is similar to that reported in other studies of cardiac devices [22, 44, 47]. However, many family members reflected that they would have liked to have more discussion regarding the risks and alternatives to getting a cardiac device. Further studies will evaluate the experiences of dyads that included patients with CI who were eligible for cardiac devices, but chose not to receive them.

Patients with AD and MCI endorsed more regret on the DRS, although the sample size limits our confidence in statistical comparisons. DRS scores for family members in the present study are comparable to what has been published in the literature for family caregivers who are involved in a decision for a family member with AD and who do not receive any support in the decision [48]. Whereas no differences in decisional conflict were observed in the patients irrespective of cognitive status, differences were noted in the family members. Among the family members of patients without CI who reported higher decisional conflict, they also reported being less certain about the risks and benefits at the time of implant compared to those family members of patients with CI. These findings are likely driven by the differences among the role of the caregiver in their role in medical decision-making (i.e., active vs. shared/passive). Although these results are counter to what we found regarding decisional regret and to what we hypothesized, they are similar to what has been reported in the literature for surrogate decision makers who do not receive any support for decision-making [49].

In the present study, patients received a pacemaker or ICD for a variety of reasons, including sinus node dysfunction, primary or secondary prevention of sudden cardiac death, and atrioventricular block. It is, therefore, possible that the decisions surrounding receipt of these devices may differ by device or indication. For example, primary prevention ICD is used to prolong life without improving QOL whereas a pacemaker for sinus node dysfunction could not only prolong life, but also improve QOL if the patient was experiencing syncope, fatigue, or palpitations. Moreover, the decision to receive a pacemaker for symptomatic bradycardia may be relatively straightforward, particularly in a patient with MCI, as compared with the decision to implant a primary prevention ICD without cardiac resynchronization therapy (CRT) in an asymptomatic patient, which poses considerable tradeoffs. Thus, further studies with larger cohorts are necessary to explore the decision-making experiences of patient and caregiver dyads by cardiac device type.

In addition to the small sample size, the present study is limited by the single site data and lack of racial or ethnic diversity in the sample, which have been shown by others to impact receipt of device [50]. In addition, for those patients who died following the procedure, the cause of death was unknown. Furthermore, the time since receiving the cardiac implant was considerable for some dyads (up to 11 years); thus, there may have been recall bias as well as survival bias, particularly for those who received a dual chamber pacemaker or ICD. Finally, there was no medical record data regarding the incidences of cardioversion or adverse events.

In conclusion, although the decision to receive a cardiac device in a person with AD requires unique considerations about the possible trade-offs of quality versus quantity of life, the patients’ cognitive status was largely not discussed with their family or physician. Most patients and family members described the procedure as “necessary” to improve the patient’s QOL, but some patients with CI had expressed regret or questioned whether the decision was “right.” Both patients and family members expressed a wish to have more information regarding the risks, benefits, and alternatives, and a majority of family members could envision a time when the patient’s cognitive and functional impairment would be too severe to get a device and acknowledge that they would focus on QOL. Multimorbidity is the new normal in our aging society, and thus medical decision-making will increasingly involve trade-offs between various lifespan and QOL-increasing interventions, which have implications for how we prefer to live and die. A better understanding of medical decision-making in these circumstances is critical to assist patients in making decisions that are right for them.

## Figures and Tables

**Table 1:** Characteristics of patients and caregivers

	Patient n=15	Caregiver n=15
Age*, years mean (SD)	80.9 (5.1)	
**Sex**, n (%)		
Male	9 (60)	3 (20)
Female	6(40)	12 (80)
**Race**, n (%)		
White	15 (100)	15 (100)
**Relationship to the patient, n(%)**		
Spouse		8 (53.3)
Adult child or child-in-law		7 (46.7)
**Cognitive status of patient**,* n (%)		
AD	8 (53.3)	
Mild cognitive impairment	2 (13.3)	
Not impaired	5 (33.3)	
**MMSE Score**,* mean (SD)	24.7 (5.1)	
**FAQ score**,* mean (SD)	6.4 (7.9)	
**Device**		
Pacemaker, n (%)	9 (60)	
Right ventricular only	2	
Right atrial and right ventricular	7	
ICD, n(%)	6 (40)	
Right ventricular only	2	
Right atrial and right ventricular	3	
Right atrial, right ventricular and left ventricular	1	
**Indications for the device**		
Sinus node dysfunction	6 (40)	
Primary prevention of Sudden Cardiac Death	4 (26.7)	
Atrioventricular block	3 (20)	
Secondary prevention of Sudden Cardiac Death	2 (13.3)	
**Indications for device therapy expressed in the standard ACC/AHA Classification format^±^**		
I	12 (80)	**-**
II	0	**-**
IIa	0	**-**
IIb	3 (20)	**-**
III	0	**-**
**Role in the decision to get a cardiac device**^‡^**,** n (%)		
Active or Shared	5 (71.4)	11 (73.3)
Passive	2 (28.6)	4 (26.7)

* Values at the time of device implant

† Class I: Conditions for which there is evidence and/or general agreement that a given procedure or treatment is beneficial, useful, and effective.

Class II: Conditions for which there is conflicting evidence and/or a divergence of opinion about the usefulness/efficacy of a procedure or treatment.

Class IIa: Weight of evidence/opinion is in favor of usefulness/efficacy.

Class IIb: Usefulness/efficacy is less well established by evidence/opinion.

Class III: Conditions for which there is evidence and/or general agreement that a procedure/treatment is not useful/effective and in some cases may be harmful.

**Table 2:** Themes and Quotations

Theme	Quotation number	Respondent information	Representative Quotation
**Limited influence of the patient’s cognitive status in medical decision-making**	1	Spouse of a patient with AD who received ICD	“Oh yeah, [telling the doctor she has AD] that’s one of the first things I mention to all the doctors. In fact, they had a sitter several days with her [while in the hospital]. That’s one of the first things I mention to medical people. No one mentioned her AD when we discussed the pacemaker.”
	2	Family member of a patient who had MCI at the time of their implant that progressed to AD at the time of the interview who received ICD	“Once the [device] is in there, if you have other kinds of medical problems, it could keep you going kind of artificially rather than letting your life end in a natural kind of way. Just think about that. Is that what you want?”
	3	Patient without CI who received pacemaker	“Unless I am so debilitated, so sick, I don’t want to continue, but I can’t envision that at the moment.”
**Circumstances around the decision to get the device**	4	Daughter of a patient with AD who received a pacemaker	“I believed it was urgent. It was a priority according to the doctors. It wasn’t something that you could debate.”
	5	Spouse of a patient with MCI who received an ICD for primary prevention of sudden cardiac death	“Well, the doctor strongly advised it because he was [in] atrial fib, and so he advised us then to put it in. He just presented to us what difference it would make in him and, you know, that he’d have to feel better afterwards because it [the atrial fib] was weakening his heart. He really highly recommended that we do it.”
	6	Family member of a patient without CI who received a pacemaker	“My role was to see what he [the patient] decides and to discuss with it him. Discuss the options, and let him make the decision.”
	7	Patient without CI at the time of ICD implant but who had progressed to MCI at the time of the interview	“I didn’t want it then, and I don’t want it now. I think the whole thing was ridiculous.”
	8	Spouse of a patient without CI who received an ICD	“On that [ICD] decision that was made – I don’t worry about it anymore. I think I made the right one. Even if it wasn’t the right one, it seems to be working out.”
	9	Patient without CI and an ICD	“I’m not sure I had a choice. My thing is, now that I look back, I felt that, if this is necessary, let’s do it.”
	10	Patient without CI who received a pacemaker	“They [the doctors] just suggested. It was almost like that is the way they thought it should be done. I mean, that I should have the pacemaker, and I went along with it, because I thought that is probably what I needed.”
	11	Adult child of a patient with AD who received pacemaker	“Obviously if it would prolong her life and preserve her QOL by doing it, then I would go for it. But if all the surgery would do is maybe prolong her life, but if she no longer had any QOL, then I would be reluctant to do it.”
**Risks, benefits and long-term implications of getting a cardiac device**	12	Adult child of a patient with MCI that received an ICD	“When something like this happens, I think you don’t know what to ask. You’re just not informed enough as a layperson. We have no experience with this kind of thing, so you don’t even know what kind of questions you should ask, and if you should do something differently… Little things like how is that going to affect him, or how much does it cost, or can it wait… I don’t think we knew enough about what to ask.”
	13	Family member of a patient with AD who received ICD	“I would want to know how does it work; what are some of the side effects; what are the long-range aspects; can it keep someone alive; if they happened to go into a vegetative state, is it something that would cause someone to live longer than what they would normally want to live in a bad situation?”
